# Phytolacca Poison in Cattle

**Published:** 1902-07

**Authors:** G. R. White

**Affiliations:** Nashville, Tenn.


### PHYTOLACCA POISON IN CATTLE.

By G. R. White, D.V.S.,
NASHVILLE, TENN.

On May 20th I was called by 'phone from Winchester, a small town, the county-seat of Franklin County, Tennessee, situated eighty-five miles south of Nashville, at the foot of the Cumberland Mountain range.

The person at the other end of the 'phone informed me that five of his best young cattle out of a herd of thirteen were affected with some peculiar malady which in his as well as in his neighbors' opinion was a contagious disease of some character. I asked him to describe to me all noticeable symptoms, which he did, as follows:

"Without any apparent cause my cattle ceased eating four days ago, and are now in a 'bad fix.' I have five sick out of a herd of thirteen. They were at first constipated, but are now passing much mucus, together with clots of blood from the bowel. I also notice some shreds of intestine. They are lifeless, noses almost against the ground, ears flopped, eyes sunken, back arched, high fever, muzzle dry and hot, slight discharge from nose; almost constant straining in their endeavor to pass feces from the rectum. They are weak, and on this account stagger and stumble whenever they attempt to walk. Complete loss of appetite and cessation of rumination." I informed him that it would be impossible for me to make a diagnosis with any degree of certainty unless he could arrange in some way for me to see the cattle as well as the pasture upon which they had been grazing previous to the attack. He then told me to come at once and make an investigation. Upon my arrival I found the cattle suffering as he had described.

I pronounced the disease hemorrhagic enteritis, accompanied by dysentery, and began at once to investigate the cause. I questioned the owner in regard to the character of food the cattle had been eating. He informed me that they had been running at grass for the past few months, and to his personal knowledge had eaten nothing except what was obtained from pasture. I asked to be shown the pasture, which was a seventy-five-acre field, with plenty of grass and clover of a good quality. We searched this pasture from end to end, but failed to find anything that, in my opinion, would produce the trouble with which his cattle were suffering. We gave up in despair the idea of locating the trouble, and were returning to the barn, when the owner, for some unexplainable reason, informed me that he had allowed the cattle to run for two days on "a winter oat patch" which had been cleared of timber the year before ("new ground"). We visited this "patch," and found thousands of phytolacca plants ("poke stalk"). Hundreds of these plants had been eaten off even with the ground by the cattle, so my diagnosis was hemorrhagic enteritis, accompanied by dysentery, due to poison from eating phytolacca plants.

